# Succinate and Lactate Production from *Euglena gracilis* during Dark, Anaerobic Conditions

**DOI:** 10.3389/fmicb.2016.02050

**Published:** 2016-12-21

**Authors:** Yuko Tomita, Kazumasa Yoshioka, Hiroko Iijima, Ayaka Nakashima, Osamu Iwata, Kengo Suzuki, Tomohisa Hasunuma, Akihiko Kondo, Masami Yokota Hirai, Takashi Osanai

**Affiliations:** ^1^School of Agriculture, Meiji UniversityKawasaki, Japan; ^2^euglena Co., LtdTokyo, Japan; ^3^Graduate School of Science, Technology and Innovation, Kobe UniversityKobe, Japan; ^4^RIKEN Center for Sustainable Resource ScienceYokohama, Japan

**Keywords:** biorefinery, *Euglena*, lactate, microalgae, succinate

## Abstract

*Euglena gracilis* is a eukaryotic, unicellular phytoflagellate that has been widely studied in basic science and applied science. Under dark, anaerobic conditions, the cells of *E. gracilis* produce a wax ester that can be converted into biofuel. Here, we demonstrate that under dark, anaerobic conditions, *E. gracilis* excretes organic acids, such as succinate and lactate, which are bulk chemicals used in the production of bioplastics. The levels of succinate were altered by changes in the medium and temperature during dark, anaerobic incubation. Succinate production was enhanced when cells were incubated in CM medium in the presence of NaHCO_3_. Excretion of lactate was minimal in the absence of external carbon sources, but lactate was produced in the presence of glucose during dark, anaerobic incubation. *E. gracilis* predominantly produced L-lactate; however, the percentage of D-lactate increased to 28.4% in CM medium at 30°C. Finally, we used a commercial strain of *E. gracilis* for succinate production and found that nitrogen-starved cells, incubated under dark, anaerobic conditions, produced 869.6 mg/L succinate over a 3-day incubation period, which was 70-fold higher than the amount produced by nitrogen-replete cells. This is the first study to demonstrate organic acid excretion by *E. gracilis* cells and to reveal novel aspects of primary carbon metabolism in this organism.

## Introduction

*Euglena gracilis* is a eukaryotic, unicellular phytoflagellate in the genus *Euglena*, characterized by dynamic and flexible cell structure, and commercially cultivated as a nutritional food. *E. gracilis* has also been studied as a biocatalyst for the production of bioenergy. *E. gracilis* cells fix carbon dioxide (CO_2_) and produce the polysaccharide paramylon, a β-1,3-glucan (Calvayrac et al., 1981). Paramylon stored under aerobic conditions is degraded during anaerobic cultivation to produce a wax ester consisting of saturated fatty acids and alcohol chains (Inui et al., 1984). Wax esters act as electron sinks from glycolytic ATP and pyruvate oxidation under anaerobic conditions (Hoffmeister et al., 2005). Under aerobic conditions, fatty acid biosynthesis starts with pyruvate and acetyl-CoA in the mitochondria (Inui et al., 1984); therefore, pyruvate and acetyl-CoA metabolism varies widely depending on the growth conditions. Adding acetate or ethanol into the medium increases the activities of acetate kinase, malate synthase and isocitrate lyase, suggesting that acetate is assimilated and metabolized through the glyoxylate pathway (Woodward and Merrett, 1975; Nakazawa et al., 2005). External ethanol is metabolized by mitochondrial alcohol dehydrogenase, which can assimilate other alcohols such as 1-butanol and 1-heptanol (Ono et al., 1995). Thus, the metabolism of pyruvate, acetyl-CoA, and organic acids in the tricarboxylic acid (TCA) cycle is dramatically changed by the growth conditions. The effect of external metabolites on primary carbon metabolism inside the cells is determined by several groups. However, the excretion of metabolites from carbon metabolism has only been detailed in the wax ester.

Succinate production has been intensively evaluated in heterotrophic bacteria because of their importance in biorefinery. Succinate is used as a building block for various compounds including the widely used plastic polybutylene succinate. Succinate is produced petrochemically, but bio-succinate production is preferable for reducing the environmental burden, as proposed by the United States Department of Energy (Werpy and Petersen, 2004). Succinate production using heterotrophic bacteria requires external carbon sources such as glucose; however, direct conversion of CO_2_ is preferable to avoid competition with foods. Recently, it was found that several cyanobacteria excrete organic acids under dark, anaerobic conditions (McNeely et al., 2014; Osanai et al., 2015; Iijima et al., 2016; Ueda et al., 2016). Cyanobacterial cells accumulate glycogen via fixation of CO_2_ under photoautotrophic conditions and glycogen is degraded under dark, anaerobic conditions. These compounds are converted into organic acids, such as lactate, acetate, and succinate, which are excreted under dark, anaerobic conditions (McNeely et al., 2014; Osanai et al., 2015; Hasunuma et al., 2016; Iijima et al., 2016; Ueda et al., 2016). Recently, succinate levels have reached 430 mg/L by metabolic engineering of cyanobacteria (Lan and Wei, 2016). This process is known as autofermentation and the organic acids are produced from CO_2_ by cyanobacteria as biocatalysts.

In this study, we found that the cells of *E. gracilis* excrete succinate under dark, anaerobic conditions. Lactate was also excreted from the cells following the addition of glucose, revealing valuable chemical production by *E. gracilis*.

## Materials and Methods

### *Euglena* Strains and Culture Conditions

Two strains of *E. gracilis*, NIES-48 from the National Institute for Environmental Studies, Japan and commercial strain from euglena, Co., Ltd, (Tokyo, Japan) (Yamada et al., 2016) were used in this study. *E. gracilis* cells were grown in modified CM medium (adjusted to pH 3.5) (Cramer and Myers, 1952). For preculture, cells were grown in liquid medium bubbled with 1% (v/v) CO_2_ in air and incubated in a plant growth chamber (TOMY, Tokyo, Japan) at 25°C under 12 h light/12 h dark conditions with white light at ∼40 μmol photons m^-2^ s^-1^. After preculture, the cells were inoculated into 5 L of modified CM medium containing 10 mM NaHCO_3_ in an acrylic tank at room temperature (approximately 25°C) and cultivated for approximately 15 days. Cell cultures were mixed with a magnetic stirrer and exposed to white light using LED at 60 μmol photons m^-2^ s^-1^. For nitrogen depletion, (NH_4_)_2_HPO_4_ was replaced with KH_2_PO_4_. Cell densities were measured at *A*_730_ using a Shimadzu UV-2400 spectrophotometer (Shimadzu, Kyoto, Japan).

### Dark, Anaerobic Incubation

Dark, anaerobic incubation was performed as described previously (Osanai et al., 2015) with modifications. Cells were concentrated in 10 mL of modified CM medium or HEPES buffer (20 mM HEPES-KOH, pH 7.8) at *A*_730_ = 20 in a GC vial; equal volumes of cultures were concentrated when using the commercial strain. The vials were sealed with a butyl rubber cap and nitrogen gas was introduced using syringes for 1 h. Anaerobic conditions were maintained by removing the syringes and wrapping the vials with aluminum foil, followed by shaking at 25 or 30°C for 3 days. After cultivation, the cell cultures were centrifuged at 5800 × *g* for 2 min, the supernatant was filtered, and 1 mL of supernatant was freeze-dried for 1 day. The pH of the supernatants was measured using a LAQUAact pH meter (Horiba, Kyoto, Japan). Organic acids in the dried sample were analyzed by high performance liquid chromatography (HPLC). For the experiment using the commercial strain, cell cultures were concentrated from the volumes of 200 or 800 mL to those of 10 mL in HEPES buffer and similarly incubated under dark, anaerobic conditions; excreted organic acids were then measured using HPLC.

### Measurement of Excreted Organic Acids Using HPLC

Organic acids were analyzed as described previously (Osanai et al., 2015). Freeze-dried supernatants were resolved in 100 μL of filtered 3 mM perchloric acid. The resolved samples were analyzed by HPLC using an LC-2000Plus System (JASCO, Tokyo, Japan) with a photodiode array detector and two RSpak KC-811 columns (Showa Denko, Tokyo, Japan). Organic acids were quantified using 0.2 mM bromothymol blue in 15 mM sodium phosphate buffer; peaks were detected at 445 nm. The temperature of the column was 60°C, and flow rates of 3 mM perchloric acid and 0.2 mM bromothymol blue solutions were 0.7 and 1.2 mL/min, respectively.

### Determination of Proportion of Lactate Isomers

Lactates were quantified using the F-Kit D-lactate/L-lactate (JK International, Tokyo, Japan) according to the manufacturer’s instruction. Fifty microliters of the supernatant was mixed with 610 μL glycylglycine buffer (pH 10.0) containing 38.4 mM glutamate, 22.3 mM NAD, 15.7 U glutamate-pyruvate transaminase solution and sterilized distilled water for a total volume of 1 mL. Absorbance was measured at 340 nm before and after the addition of 20 μL of L-lactate or D-lactate dehydrogenase for 5 min (final concentrations of L-/D- lactate dehydrogenases were 108 U).

## Results

### Cultivation of *E. gracilis* Cells and Succinate Production

**FIGURE 1:**
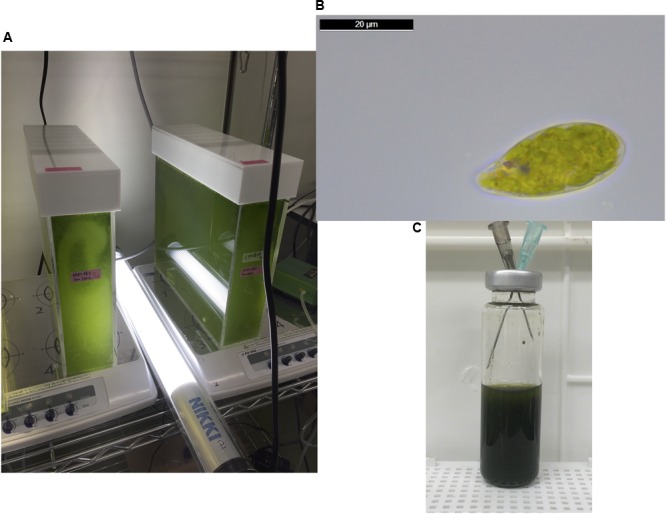
**(A)** The cells of *Euglena gracilis* were cultured at approximately 25°C in a 5 L acrylic tank placed on a magnetic stirrer under an LED light with an intensity of 60 μmol photons m^-2^ s^-1^. **(B)** A cell of *E. gracilis* grown under photoautotrophic conditions, observed using a Leica DM500 microscope (Leica Microsystems, Tokyo, Japan). The image was analyzed using the Leica Application Suite software, version 4.6. **(C)** Anaerobic incubation for excretion of organic acid. Cells were concentrated in 10 mL CM medium or HEPES buffer at *A*_730_ = 20 in a 20 mL GC vial. The vial was sealed with a butyl rubber cap and nitrogen gas was introduced using syringes for 1 h. After removing the syringes, the vial was wrapped with aluminum foil and incubated for 3 days with shaking at 25 or 30°C.

**FIGURE 2:**
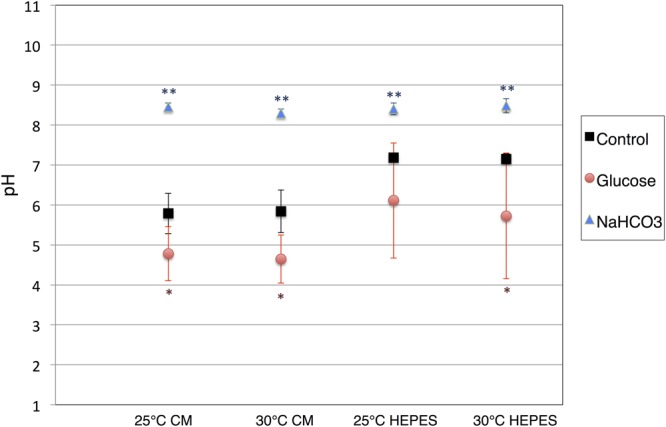
**pH of the supernatants after dark, anaerobic incubation**. The cells of *E. gracilis* were incubated under dark, anaerobic conditions for 3 days in CM medium or HEPES buffer with/without 100 mM glucose and 100 mM NaHCO_3_. After centrifugation to remove the cells, the pH of the supernatants was quantified. Data represent the mean ± SD from biologically independent samples (*n* = 5–7). Asterisks indicate statistically significant differences in pH from supernatants collected from cells grown with and without external carbon sources (Student’s *t*-test; ^∗^*P* < 0.05, ^∗∗^*P* < 0.005).

The cells of *E. gracilis* NIES-48 were grown in a 5 L acrylic tank placed on a magnetic stirrer (**Figure 1A**). Cells were grown at room temperature (approximately 25°C) under white light from an LED at 60 μmol photons m^-2^ s^-1^. Organic acid excretion from *E. gracilis* cells was assessed as described previously for cyanobacteria (Osanai et al., 2015). Cells grown under photoautotrophic conditions (**Figure 1B**) were concentrated in CM medium (pH 3.5) or HEPES buffer (pH 7.8) in a GC vial (**Figure 1C**), and incubated under dark, anaerobic conditions for 3 days at 25 and 30°C. Additionally, 100 mM glucose or 100 mM NaHCO_3_ was added as a source of carbon during the incubation. In the absence of external carbon sources, the pH of the supernatant, after a 3-day incubation in CM medium and HEPES buffer, was 5.8 and 7.2, respectively, regardless of the temperature (**Figure 2**). The addition of glucose reduced the pH by 0.9–1.5 in both CM medium and HEPES buffer (**Figure 2**). The addition of NaHCO_3_ increased the pH to 8.3–8.5 under all four conditions (**Figure 2**).

**FIGURE 3:**
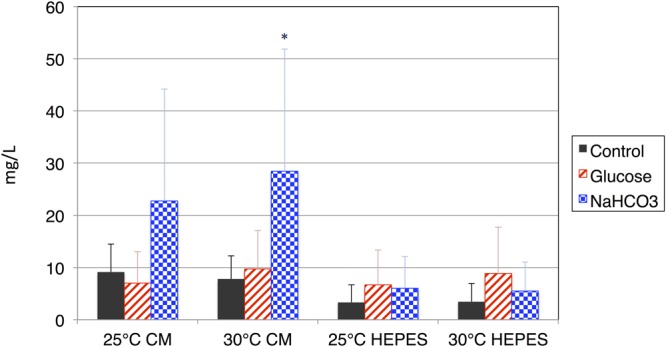
**Levels of succinate produced by cells of *E. gracilis* incubated under dark, anaerobic conditions.** The cells of *E. gracilis* were incubated under dark, anaerobic conditions for 3 days in CM medium or HEPES buffer with/without 100 mM glucose or 100 mM NaHCO_3_; the levels of succinate excreted from the cells were quantified using HPLC. Data represent the mean ± SD from biologically independent samples (*n* = 6–10). Asterisk indicates statistically significant differences in the levels of succinate produced with and without external carbon sources (Student’s *t*-test; ^∗^*P* < 0.05).

Organic acids excreted from the cells were quantified using HPLC, and succinate was detected in the supernatant after dark, anaerobic incubation. Overall, the levels of succinate excreted from the cells incubated in CM medium were higher than those from cells incubated in HEPES buffer at both 25 and 30°C (**Figure 3**). The addition of glucose increased the levels of succinate in HEPES buffer, but decreased those in the CM medium (**Figure 3**). The addition of NaHCO_3_ increased the levels of succinate under all four conditions, regardless of the medium and temperature (**Figure 3**). The maximum concentration of succinate obtained from cells incubated in the CM medium containing NaHCO_3_ at 30°C was 28.4 mg/L.

### Lactate Production

**FIGURE 4:**
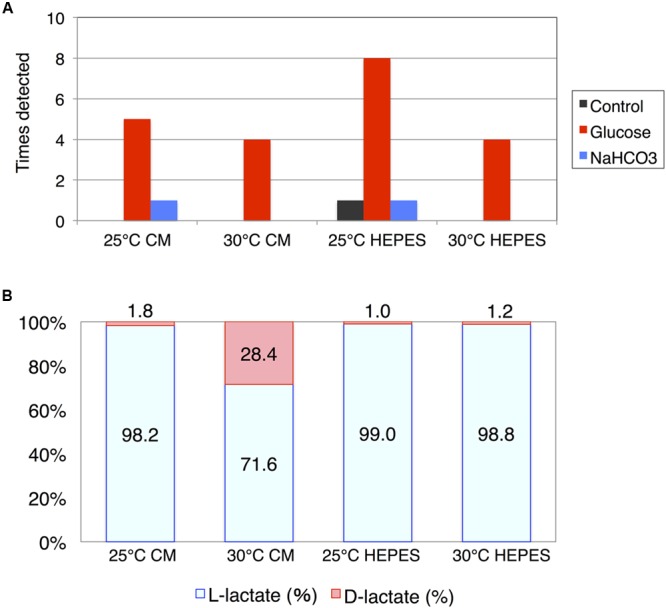
**Lactate production by cells of *E. gracilis* incubated under dark, anaerobic conditions. (A)** The number of times lactate was detected using HPLC. The cells of *E. gracilis* were incubated under dark, anaerobic conditions for 3 days in CM medium or HEPES buffer with/without 100 mM glucose and 100 mM NaHCO_3_. Experiments were repeated 10 times and the number of times lactate was detected at more than 10 mg/L was counted and visualized. **(B)** Percentage of L-lactate and D-lactate. Lactates from *E. gracilis* cells incubated under dark, anaerobic conditions for 3 days in CM medium or HEPES buffer containing 100 mM glucose were analyzed. Two replicates of each sample, containing the highest level of lactate, were used to determine chirality.

In addition to succinate, HPLC analysis revealed that lactate was produced by the cells of *E. gracilis* during dark, anaerobic incubation. In contrast to succinate, lactate production showed poor reproducibility. The experiment was repeated 10 times and the number of experiments in which lactate was produced at a concentration greater than 10 mg/L was counted. Lactate was minimally detected when the cells were incubated under dark, anaerobic conditions without external sources of carbon (**Figure 4A**). However, lactate was produced in the presence of glucose as an external carbon source. Lactate production was observed eight times when cells were incubated in HEPES buffer at 25°C (**Figure 4A**). NaHCO_3_ had a weaker effect on lactate production compared with that of glucose (**Figure 4A**). The highest level of lactate was, produced by cells incubated in HEPES buffer containing 100 mM glucose at 25°C, was 1.4 g/L.

Because lactate is chiral, we enzymatically determined the ratio of L-/D-lactate produced from *E. gracilis* cells incubated in the presence of glucose; we used two replicates of each sample containing the highest levels of lactate. While both L-lactate and D-lactate enantiomers were detected; L-lactate predominated (**Figure 4B**). However, when the cells were incubated in CM medium at 30°C under dark, anaerobic conditions, the percentage of D-lactate was increased to 28.4% (**Figure 4B**).

### Succinate Production Using Commercial Strain of *E. gracilis*

**FIGURE 5:**
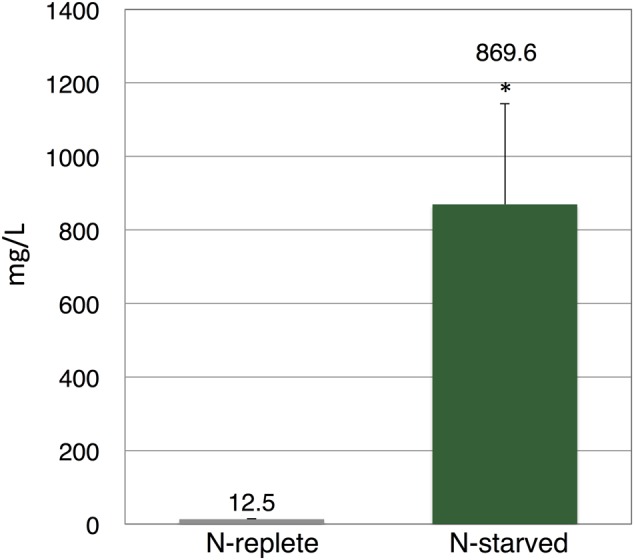
**Levels of succinate produced by commercial strain of *E. gracilis*.** Cells were cultured under nitrogen-replete or nitrogen-starved cells for 11 days, then incubated under dark, anaerobic conditions for 3 days in HEPES buffer. Excreted succinate was quantified using HPLC. Data represent the mean ± SD of three biologically independent samples. Asterisks indicate statistically significant differences between the two samples (Student’s *t*-test; ^∗^*P* < 0.05).

Finally, to evaluate the applicability of our method, we used the commercial strain of *E. gracilis*, which is derived from NIES-48 and has been commercially cultivated by the euglena, Co., Ltd, for 10 years (Yamada et al., 2016). To precisely control the growth conditions, the cells were cultivated in a plant growth chamber at 25°C under white light exposure (12 h light/12 h dark). The production of wax ester is enhanced by nitrogen starvation before dark, anaerobic cultivation (Arashida and Mitra, 2011). For nitrogen starvation, cells were cultivated for 11 days in nitrogen-depleted medium. Then, nitrogen-replete and nitrogen-starved cells, each in a volume of 800 mL culture medium, were concentrated and incubated under dark, anaerobic conditions. To reduce cost, all anaerobic incubation was performed in HEPES buffer without external carbon sources. The succinate level from nitrogen-replete cells was 12.5 mg/L (**Figure 5**). Succinate levels from nitrogen-starved cells reached 869.6 mg/L, which was 70-fold higher than that from nitrogen-replete cells (**Figure 5**). Addition of external carbon sources or potassium, which enhanced succinate production in *Synechocystis* sp. PCC 6803 (Ueda et al., 2016), did not increase succinate levels from nitrogen-starved cells (Supplementary Figure S1).

## Discussion

**FIGURE 6:**
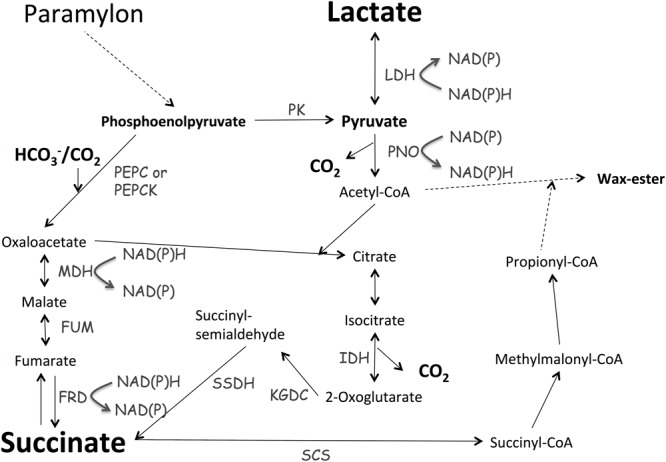
**Metabolic map describing lactate and succinate production.** Metabolic map was generated based on the map described by Padermshoke et al. (2016). MDH, malate dehydrogenase; FRD, fumarate reductase; FUM, fumarase; IDH, isocitrate dehydrogenase; KGDC, α-ketoglutarate dehydrogenase; LDH, lactate dehydrogenase; PEPC, phosphoenolpyruvate carboxylase; PEPCK, phosphoenolpyruvate carboxykinase; PK, pyruvate kinase; PNO, pyruvate:NADP^+^ oxidoreductase; SSDH, succinyl-semialdehyde dehydrogenase.

In this study, we found that *E. gracilis*, transferred from light, aerobic conditions to dark, anaerobic conditions, excreted succinate and lactate during incubation. These metabolites are typical fermentative products excreted under dark, anaerobic conditions to oxidize NAD(P)H for substrate-level phosphorylation. Controlling growth conditions is important for biochemical production using *E. gracilis* (Grimm et al., 2015). The levels of α-tocopherol in cells grown under photoautotrophic conditions are 1.6-fold higher than those in cells grown under heterotrophic conditions (Grimm et al., 2015). The levels of paramylon in cells grown heterotrophically are 12-fold higher than those in cells grown photoautotrophically (Grimm et al., 2015). Adding glucose and ethanol into the medium increases paramylon production in *E. gracilis* (Rodríguez-Zavala et al., 2010). Here, we showed that changing the medium and temperature under dark, anaerobic conditions altered the levels of succinate from *E. gracilis* (**Figure 3**). Lactate production was dependent on supplementation with external glucose, and chirality was altered temperature and type of medium used (**Figure 4B**). The poor reproducibility of lactate production indicates that lactate excretion and biosynthesis are controlled by several factors in addition to temperature and type of medium (**Figure 4A**). Because the genomic sequence of *E. gracilis* has not been determined, the number of lactate dehydrogenases is unknown. The activity of L-lactate and D-lactate dehydrogenase has been shown in the mitochondria and cytosol of *E. gracilis* (Jasso-Chávez et al., 2001, 2005). Thus, the production of both isomers using *E. gracilis* is rational; nevertheless the physiological relevance requires further examination. In the case of *Lactobacillus coryniformis*, L-lactate dehydrogenase is more stable under heat treatment than D-lactate dehydrogenase is; this stability results in increased levels of L-lactate at higher fermentation temperature (Gu et al., 2014). Thus, thermostabilities of lactate dehydrogenases determine the ratio of L-lactate/D-lactate. Analysis of lactate dehydrogenases in *E. gracilis* shows that D-lactate dehydrogenase is more tolerant to high temperature than L-lactate dehydrogenase is (Jasso-Chávez et al., 2001), which is consistent with our result (**Figure 4B**). The production of lactate and succinate requires oxidation of NAD(P)H for substrate-level phosphorylation (**Figure 6**). As shown in the metabolic map, succinate production requires additional carbon fixation by PEPC/PEPCK, while lactate production does not (**Figure 6**). This may be the reason for increased lactate production in the presence of glucose (**Figure 4A**).

Recently, two independent groups analyzed the transcriptome of *E. gracilis* (O’Neill et al., 2015; Yoshida et al., 2016) and found that *E. gracilis* contains approximately 50,000 unique transcripts; 800 and 1280 transcripts were upregulated and downregulated by a 24-h anaerobic incubation, respectively (Yoshida et al., 2016). The expression of genes involved in photosynthesis, nucleotide metabolism, TCA cycle, and oxidative phosphorylation was downregulated under anaerobic conditions (Yoshida et al., 2016). Yoshida et al. (2016) proposed *E. gracilis* uses fumarate, rather than O_2_, as an electron acceptor under anaerobic conditions; this agrees with our results showing that *E. gracilis* produced succinate during dark, anaerobic incubation (**Figure 3**). Metabolomic analysis, conducted using the same strain of *E. gracilis*, shows that compared with the levels of metabolites in cells grown under light, aerobic conditions, the levels of pyruvate decreased to 4% in cells grown under dark, anaerobic conditions (Matsuda et al., 2011). The authors suggest that the decrease in pyruvate was related to the biosynthesis of wax ester, which is produced from acetyl-CoA (Matsuda et al., 2011); however, we suggest that the decreased pyruvate is also related to the production of succinate and lactate under dark, anaerobic conditions (**Figures 3** and **4**). Although the biosynthetic pathway of succinate is currently unclear, in the unicellular cyanobacterium *Synechocystis* sp. PCC 6803, succinate biosynthesis under dark, anaerobic conditions begins with the conversion of phosphoenolpyruvate to oxaloacetate via phosphoenolpyruvate carboxylase (PEPC) and through the reductive TCA cycle (Hasunuma et al., 2016). In *E. gracilis*, phosphoenolpyruvate carboxykinase (PEPCK) assimilates HCO_3_^-^ under dark, anaerobic conditions (Padermshoke et al., 2016). Combined with the results of transcriptomic and metabolomic analyses (Yoshida et al., 2016), our results suggest that *E. gracilis* produces succinate via the reductive TCA cycle under dark, anaerobic conditions (**Figure 6**); to confirm this notion, metabolic flux analysis must be performed. Under dark, anaerobic conditions, dihydroxyacetone phosphate accumulates in *E. gracilis* cells (Matsuda et al., 2011), and *Synechocystis* sp. PCC 6803 (Osanai et al., 2015). Conversely, the effect of potassium on succinate production in *Synechocystis* sp. PCC 6803 differs from that in *E. gracilis* (Supplementary Figure S1; Ueda et al., 2016). Thus, comparing the results of metabolomic analyses will help to elucidate the differences in primary carbon metabolism among photosynthetic organisms grown under anaerobic conditions. Carboxylation of phosphoenolpyruvate by PEPC or PEPCK is a rate-limiting step during succinate production in heterotrophic bacteria (Millard et al., 1996; Lee et al., 2006; Tan et al., 2013). HCO_3_^-^ is a substrate of PEPCK/PEPC (**Figure 6**); therefore, the addition of NaHCO_3_ promoted succinate production in *E. gracilis*. PEPC activity in *E. gracilis* increases at the end of the exponential phase, possibly because an increase in gluconeogenesis is needed to produce paramylon (Briand et al., 1981). Therefore, the status of the cells before dark, anaerobic incubation is also important for succinate production, as was demonstrated using the commercial strain in this study (**Figure 5**). Supplementation with external sources of carbon did not increase succinate production in the nitrogen-starved cells (Supplementary Figure S1), indicating that carbon sources are not rate-limiting for nitrogen-starved cells because of high accumulation of paramylon induced by nitrogen starvation. Our results demonstrate that *E. gracilis* can be used to produce various chemicals as biocatalysts for microbial cell factories.

## Author Contributions

YT, KY, HI, and TO performed the experiments and analyzed the data, AN, OI, KS, TH, AK, and MH, designed the study, and TO wrote the manuscript.

## Conflict of Interest Statement

The authors declare that the research was conducted in the absence of any commercial or financial relationships that could be construed as a potential conflict of interest.
