# Supplementary material for: Succinate and Lactate Production from Euglena gracilis during Dark, Anaerobic Conditions
Source: Front Microbiol. 2016 Dec 21;7:2050. doi: 10.3389/fmicb.2016.02050 (PMC5174102; doi:10.3389/fmicb.2016.02050)
Supplement: Supplementary file 1 [file Presentation_1.PDF]

Figure S1. Levels of succinate produced by commercial strain of *Euglena gracilis*. Cells in 200-mL cultures were nitrogen-starved for 8 days and incubated under dark, anaerobic conditions for 3 days in HEPES buffer containing 100 mM $NaHCO_3$, 100 mM glucose, or 100 mM KCl. Excreted succinate was quantified using HPLC. Mock indicates succinate levels produced without external carbon sources or KCl. Data represent the mean ± SD of three biologically independent samples.
