# Supplementary figures and images for: Succinate and Lactate Production from Euglena gracilis during Dark, Anaerobic Conditions
Source: Front Microbiol. 2016 Dec 21;7:2050. doi: 10.3389/fmicb.2016.02050 (PMC5174102; doi:10.3389/fmicb.2016.02050)

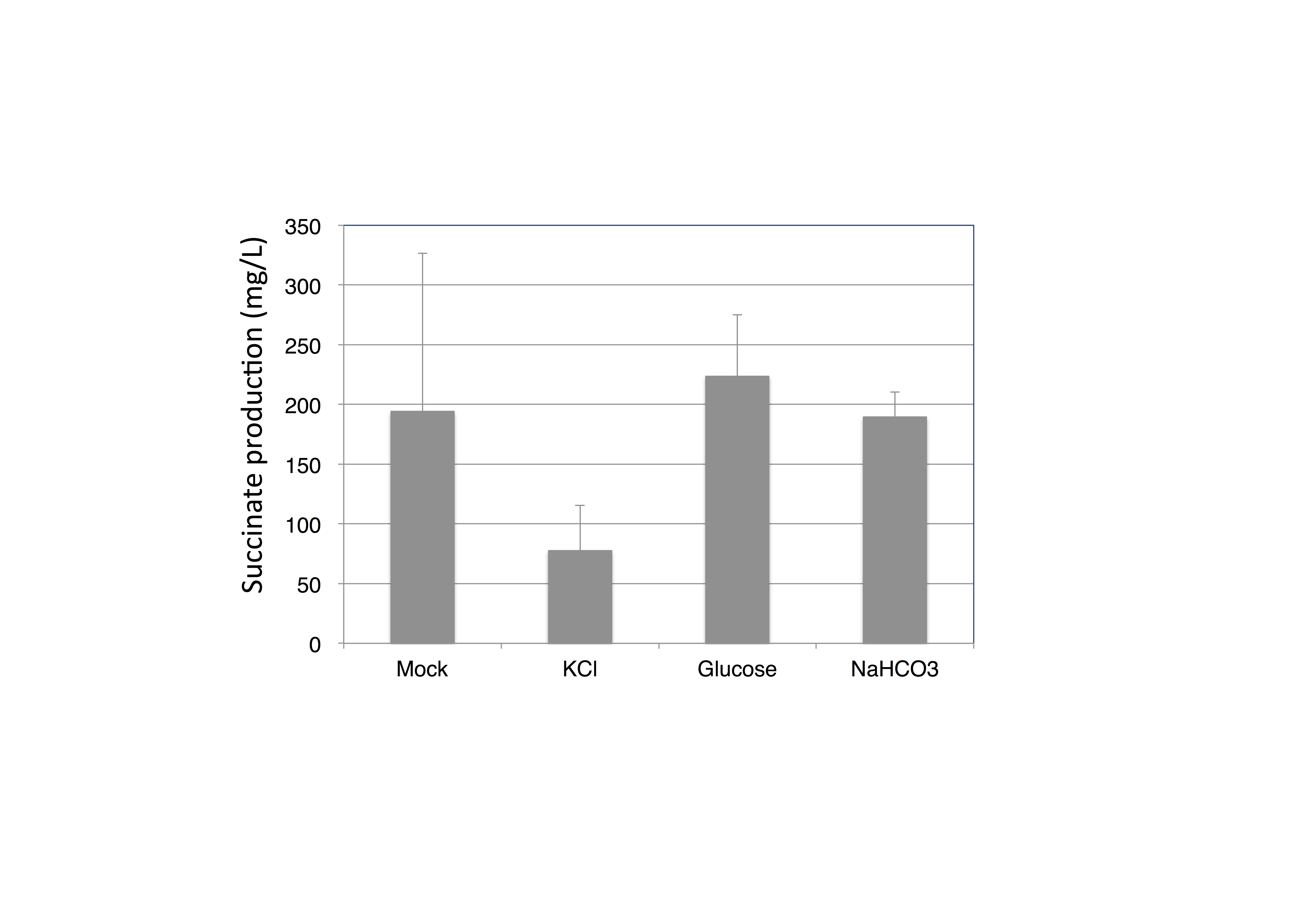

Supplement: Supplementary file 2 [file Image_1.TIFF]
